# Polarity Control of ZnO Films Grown on Ferroelectric (0001) LiNbO_3_ Substrates without Buffer Layers by Pulsed-Laser Deposition

**DOI:** 10.3390/nano10020380

**Published:** 2020-02-22

**Authors:** Im Taek Yoon, Juwon Lee, Ngoc Cuong Tran, Woochul Yang

**Affiliations:** 1 Quantum Functional Semiconductor Research Center (QSRC), Dongguk University, 26 Phildong 3ga, Chung gu, Seoul 100-715, Korea; ityoon@dongguk.edu; 2 Department of Physics, Dongguk University, 26 Phildong 3ga, Chung gu, Seoul 100-715, Korea; Cuong.Tran@onsemi.com

**Keywords:** polarity control, ZnO, ferroelectric substrate

## Abstract

For this study, polarity-controlled ZnO films were grown on lithium niobate (LiNbO_3_) substrates without buffer layers using the pulsed-laser deposition technique. The interfacial structure between the ZnO films and the LiNbO_3_ was inspected using high-resolution transmission electron microscopy (HR-TEM) measurements, and X-ray diffraction (XRD) measurements were performed to support these HR-TEM results. The polarity determination of the ZnO films was investigated using piezoresponse force microscopy (PFM) and a chemical-etching analysis. It was verified from the PFM and chemical-etching analyses that the ZnO film grown on the (+z) LiNbO_3_ was Zn-polar ZnO, while the O-polar ZnO occurred on the (-z) LiNbO_3_. Further, a possible mechanism of the interfacial atomic configuration between the ZnO on the (+z) LiNbO_3_ and that on the (-z) LiNbO_3_ was suggested. It appears that the electrostatic stability at the substrate surface determines the initial nucleation of the ZnO films, leading to the different polarities in the ZnO systems.

## 1. Introduction

The controlling of the crystal polarity in wurtzite-structure semiconductors, such as ZnO and GaN, has been enormously exploited owing to the great importance of the effects on the crystal-growth modes, material property, impurity incorporation, surface stability, and electric polarization [1,2,3,4,5,6]. Moreover, polarity-controlled wurtzite semiconductors are very useful in optoelectronic device applications, such as sensors and actuators, light emitting diode (LED)s, and optical-wavelength converters due to their unique physical properties [7,8]. In recent years, considerable efforts regarding the control of the polarity of ZnO films have been undertaken, and various methods, such as that of the template layer [9,10], the nucleation of films with a doping-derived favorable polarity [11,12], and the application of an electric bias to the substrate, have been applied [13]. The techniques that have been used for the polarity control in previous studies, however, have been very complicated due to additional processes, such as the substrate treatment, the insertion of additional layers, and the necessity regarding special equipment; furthermore, these methods create crystalline defects that lead to disordered interfaces between the ZnO, buffer layer, and substrate, and they alsocause complications for the device applications [14]. The additional growth processes, defects, and dislocations can be minimized, and the polarity can also be controlled directly in the ZnO films using different-polarity ferroelectric-LiNbO_3_ substrates. 

It is well known that closely-lattice-matched substrates are more suitable for the growth of epitaxial films. The lithium niobate (LiNbO_3_) (0001) symmetry is hexagonal with a lattice constant of 5.148 Å; therefore, the planar–lattice mismatch between ZnO and LiNbO_3_ is smaller (8.49%) than the value (15.53%) for the commonly used sapphire substrate [15]. In addition, different-polarity substrates, such as (+z) LiNbO_3_ and (-z) LiNbO_3_, are commercially available. Considering the previous perspectives, ZnO films were grown on (+z) LiNbO_3_ and (-z) LiNbO_3_ for this study to develop the polarity-control technique of the wurtzite-structure semiconductors according to the facile method. The deposition of ZnO epitaxial films without buffer layers on LiNbO_3_ (0001) substrates by pulsed-laser deposition with detailed parameters was studied [16]. Together with the growth of the ZnO films, the polarity determination of the ZnO films was investigated for various device applications. The material properties of the host material, ZnO, are sound, and many of the potential applications for optoelectronics [17,18] and spintronics [19] can be employed together with gallium nitride (GaN). A key bottleneck regarding the polarity control of ZnO films is the polarity-controlled crystallization in the facile method. To the authors’ knowledge, an exploration of the direct polarity-control growth in ZnO films for which different-polarity ferroelectric-LiNbO_3_ substrates are used has not been conducted by any research group. 

The purpose of this study is the development of a novel technique for the polarity control in ZnO systems for which different-polarity ferroelectric-LiNbO_3_ substrates are applied. ZnO thin films were grown on (+z) LiNbO_3_ and (-z) LiNbO_3_ substrates using the pulsed-laser deposition (PLD) technique. The polarity determination of the samples was then analyzed using high-resolution transmission electron microscopy (HR-TEM), atomic force microscopy (AFM), X-ray diffraction (XRD), a chemical-etching method, and piezoresponse force microscopy (PFM) measurements. Furthermore, a possible mechanism of the interfacial atomic configuration between the ZnO on the (+z) LiNbO_3_ and the ZnO on the (-z) LiNbO_3_ is suggested.

## 2. Experimentals

The ZnO films were grown on the (0001) LiNbO_3_ substrates using a fully automated PLD system (Neocera, Pioneer 180, Beltsville, VA, USA). The undoped ZnO target was prepared using a conventional solid-state reaction at 1100 °C for which pure ZnO (99.999%) powders were employed. The KrF excimer laser (*λ* = 248 nm, pulse duration = 20 ns, pulse-repetition rate *f* = 5 Hz) was used for the target ablation. The laser was irradiated with a fluence of 2.66 J/cm^2^ on the target surface. The target-substrate distance was 5 cm. The background pressure of the growth chamber was approximately 10^−8^ Torr. The ZnO films were deposited at 600 °C under an O pressure of 4 mTorr. The film was grown for 50 min with a deposition rate of ~2.2 nm/min. The XRD studies were carried out using the Rigaku XRD-DMAX Rapid X-ray diffractometer (Rigaku Co., Tokyo, Japan) for the characterization of the crystallinity and the growth orientation. HR-TEM (Tecnai G2 F30 super-twin), conducted at 300 kV, was used to investigate the interfacial structure of the ZnO/(+z) LiNbO_3_ and the ZnO/(-z) LiNbO_3_. The TEM samples were prepared according to standard cross-sectional preparation procedure with a focus ion beam and mechanical thinning. The crystal polarity of the ZnO films was determined using a chemical-etching method and PFM measurements. The samples were etched in an HNO_3_ acid of 0.01% for 30 s. After the samples were cleaned in deionized water using an ultrasonic cleaner at 40 °C for 10 min, the AFM (Bruker- Nano N8 NEOS, Bruker Corp, Billerica, MA, USA) measurements in a non-contact mode were performed to analyze the surface morphology of the ZnO films before and after the etching. A commercial AFM system equipped with PFM modules (Bruker-Nano N8 NEOS) was used to investigate the local piezo-response polarization effect of the ZnO films in the contact mode with a load force of 10 nN. For the PFM measurements, the conductive Cr/Pt-coated Si tip (Budget sensors Multi75-G, Budget sensors, Sofia, Bulgaria) with a radius of less than 10 nm. The cantilever with a spring constant of 0.2 N/m and a resonant frequency of 13 kHz was used for both imaging (modulation voltage of 4.3 V*_pp_* at 30 kHz) and for hysteresis loop measurement. For electrical connection of the samples, the backside of the samples was contacted to the AFM sample stage with carbon tape. An external AC modulation voltage superimposed a DC bias was applied between the sample and the grounded tip. The local piezoresponse loop was measured by PSIA XE-100 with a lock-in amplifier (Standard Research Systems) using the same probes. 

## 3. Results and Discussion

### 3.1. Crystal-Structure Analysis

**Figure 1:**
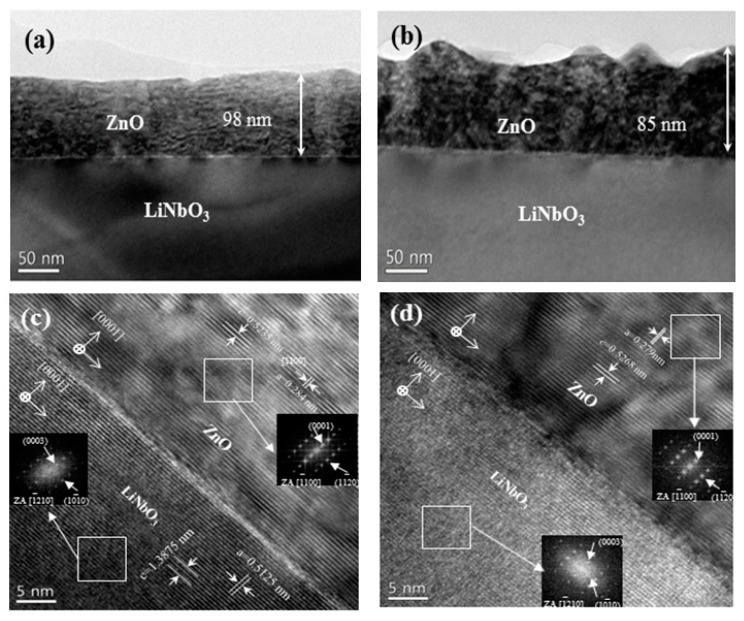
The cross-sectional using high-resolution transmission electron microscopy (HR-TEM) image of the ZnO films grown on the lithium niobate (LiNbO_3_). (**a**) The cross-sectional TEM image of the ZnO films grown on (+z) LiNbO_3_ (**a**) and on (-z) LiNbO_3_ (**b**), respectively. The magnified cross-sectional image of the HR-TEM for the ZnO films grown on (+z) LiNbO_3_ (**c**) and (-z) LiNbO_3_ (**d**), respectively. The insets of Figure 1c,d show the selected-area diffraction pattern from the ZnO films and the LiNbO_3_, respectively.

Figure 1 shows a cross-sectional HR-TEM micrograph of the ZnO films that were grown on the LiNbO_3_ for the inspection of the interfacial structure. Typical cross-sectional HR-TEM images of the ZnO films grown on (+z) LiNbO_3_ and (-z) LiNbO_3_ are illustrated in Figure 1a,b, respectively. As can be seen from the images, the surface of the ZnO films on (+z) LiNbO_3_ was smoother than that of the ZnO films on (-z) LiNbO_3_. The film thickness of the ZnO films was estimated at approximately 98 nm for the ZnO films on (+z) LiNbO_3_ and 85 nm for the ZnO films on (+z) LiNbO_3_. The interfacial structure showed drastic differences between the ZnO films grown on (+z) LiNbO_3_ and (-z) LiNbO_3_, as shown in Figure 1c,d. A clear interfacial layer with a well-ordered structure was obtained for the ZnO films grown on (+z) LiNbO_3_ (Figure 1c), while an unclear interface with a thin transition layer that was approximately 3 nm thick was formed for the ZnO films grown on (-z) LiNbO_3_ substrate (Figure 1d). These results imply that the atomic-stacking order along the growth direction of the ZnO films grown on the different-polarity LiNbO_3_ substrates is different leading to a different ZnO polarity. The insets of Figure 1c,d represents the selected-area diffraction pattern from the ZnO film on LiNbO_3_ for the examination of the lattice structure. Accordingly, the lattice was well-aligned without any distortion and the structure of the ZnO films was a single crystalline hexagon. From the results and the analysis, the orientation relationship between the ZnO and LiNbO_3_ was determined as follows:

$$\left(0001\right)ZnO∥\left(0001\right)\;{\mathrm{LiNbO}}_{3}\;\mathrm{and}\;\left[1\bar{1}00\right]ZnO∥\left[1\bar{2}10\right]\;{\mathrm{LiNbO}}_{3}$$

To support and demonstrate the above HR-TEM results, XRD measurements were carried out for the ZnO films grown on LiNbO_3_. The XRD measurements of the ZnO films grown on (+z) LiNbO_3_ and (-z) LiNbO_3_ are presented in Figure S1 (see Figure S1 of Supplementary Materials). A dominant (002) peak at ~34° is observable in Figure S1a for both of the ZnO films grown on (+z) LiNbO_3_ and (-z) LiNbO_3_. The XRD peaks at 34.17° and 34.26° indicate that the thin films were grown along the *c*-axis orientation with a highly uniform wurtzite-lattice structure. Based on these peak positions, the lattice-constant c values were calculated as 5.23 Å and 5.25 Å for the ZnO films grown on (+z) LiNbO_3_ and (-z) LiNbO_3_, respectively. These results are consistent with the measured c parameter of the HR-TEM data. Figure S1b shows the ω-rocking curves of the ZnO films grown on (+z) LiNbO_3_ and (-z) LiNbO_3_. The FWHM (full width half medium) value of the ω-rocking curve was 0.58° for the ZnO films grown on (+z) LiNbO_3_ and 0.77° for the ZnO films grown on (-z) LiNbO_3_. The FWHM variations of the ZnO (002) peaks were in agreement with the HR-TEM results regarding the interface-structure difference between the ZnO films grown on (+z) LiNbO_3_ and (-z) LiNbO_3_.

### 3.2. The Polarity Determination

**Figure 2:**
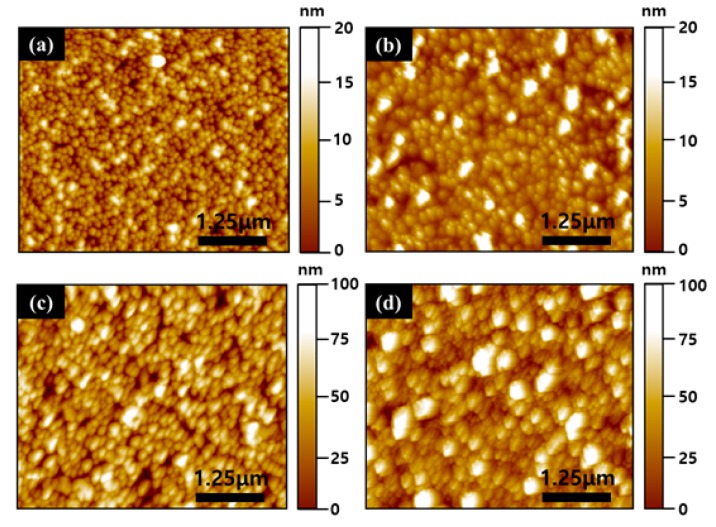
The atomic force microscopy (AFM) images of the ZnO films grown on LiNbO_3_ before and after the etching. The surface-morphology images of the ZnO films grown on (+z) LiNbO_3_ before the etching (**a**) and (-z) LiNbO_3_ before the etching (**b**). (**c**). The surface-morphology images of the ZnO films grown on (+z) LiNbO_3_ after the etching (**c**) and (-z) LiNbO_3_ after the etching (**d**).

To investigate the polarity determination of the ZnO films grown on LiNbO_3_, chemical-etching studies were carried out. Figure 2 represents the AFM images of the ZnO films grown on LiNbO_3_ before and after the etching. The ZnO films of the opposite substrate polarities showed quite different surface morphologies under the same growth condition, as shown in Figure 2a,b. The surface of the ZnO films grown on (+z) LiNbO_3_ was smoother than that of the ZnO films grown on (-z) LiNbO_3_. The surface-roughness values were 6.94 nm and 9.20 nm for the ZnO films grown on (+z) LiNbO_3_ and (-z) LiNbO_3_, respectively. The post-etching surface-morphologies of the ZnO films grown on LiNbO_3_ are shown in Figure 2c,d. The surface of the ZnO films grown on (+z) LiNbO_3_ contained pits, while a rough surface with a hillock could be observed in the ZnO films grown on (-z) LiNbO_3_ due to the rapid etching rate. To verify the previous results, the bulk-ZnO samples were also subjected to the chemical-etching experiments under the same condition, compared with the etched ZnO films (see Figure S2 in Supplementary Materials), similar results were obtained. Regarding aspects of the ZnO polarity, such as the etching rate, the roughness and shape of the etched surface, similar results have been reported [20,21]. Therefore, the results and analyses indicate that the surface of the ZnO films on (+z) LiNbO_3_ is Zn-polar, while the surface of the ZnO films on (-z) LiNbO_3_ is O-polar. 

**Figure 3:**
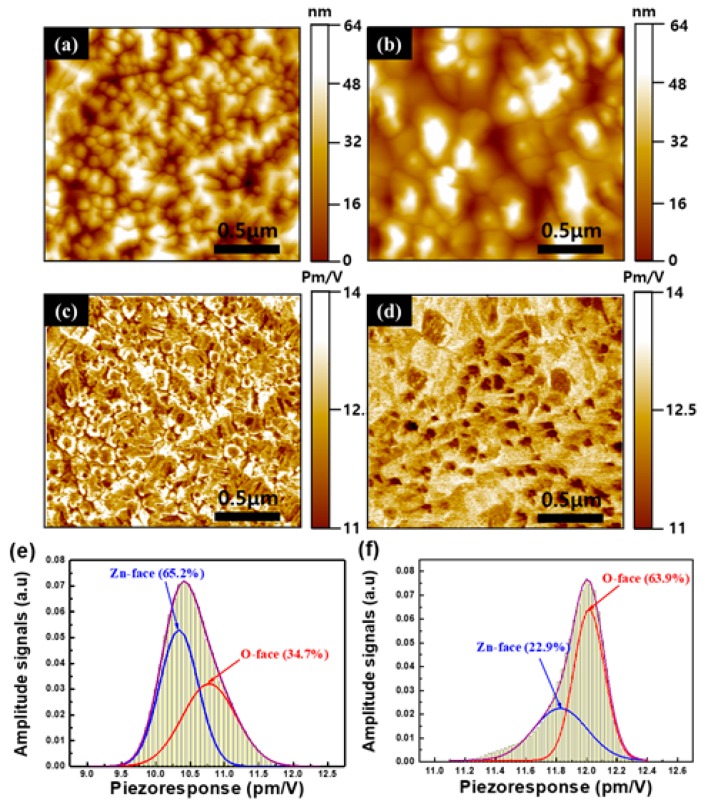
The topography, piezoresponse force microscopy (PFM)-phase images, and histograms of the ZnO films grown on (+z) LiNbO_3_ and (-z) LiNbO_3_. The topography images of the ZnO films grown on (+z) LiNbO_3_ (**a**) and (-z) LiNbO_3_ (**b**), respectively. The corresponding PFM-phase image of the ZnO films grown on (+z) LiNbO_3_ (**c**) and (-z) LiNbO_3_ (**d**), respectively. The piezoresponse histogram taken from the PFM images of the ZnO films grown on (+z) LiNbO_3_ (**e**) and (-z) LiNbO_3_ (**f**), respectively.

To gain further insight into the previous results, the PFM measurement was performed to determine the polarity of the ZnO films grown on (+z) LiNbO_3_ and (-z) LiNbO_3_. The polarity of the wurtzite semiconductors, such as ZnO and GaN, was verified through a determination of the relative phase between the modulation voltage that was applied to the material and the material deformation, which depends on the sign of the piezoelectric coefficient (*d_33_*). The results showed deformations that were in-phase regarding the metal-cation polarity and out-of-phase for the anion polarity [22,23]. The topography, PFM-phase images, and histograms of the ZnO films grown on (+z) LiNbO_3_ and (-z) LiNbO_3_ are presented in Figure 3. Figure 3a,b shows the topography images of the ZnO films grown on (+z) LiNbO_3_ and (-z) LiNbO_3_, respectively. The PFM-phase images of the ZnO films grown on (+z) LiNbO_3_ and (-) LiNbO_3_ are shown in Figure 3c,d, respectively. Most of the grains of the ZnO films grown on (+z) LiNbO_3_ exhibited a dark contrast, while most of the grains of the ZnO films grown on (-z) LiNbO_3_ displayed a bright contrast, as shown in Figure 3c,d, respectively. The contrasts of the dark and bright domains correspond to the grains with the downward and upward spontaneous-polarization orientations, respectively. These results mean that the spontaneous polarization (*P_s_*) in most of the grains for the ZnO films on (+z) LiNbO_3_ is downward, whereas the spontaneous-polarization orientation of most of the grains for the ZnO films on (-z) LiNbO_3_ is upward. To determine the statistical distribution of the local polarization in the ZnO films grown on the LiNbO_3_, the frequency of the local piezoresponse on the overall surface could be obtained from PFM images analysis using image-analysis software (SPIP) in the AFM system since the number of the points (pixels) on the PFM image corresponds to a given piezoresponse signal. Figure 3e,f represents the piezoresponse histograms that were taken from the PFM images of Figure 3c,d, respectively. Figure 3e shows the left-skewed histogram of the PFM image in the dark contrast of Figure 3c. From the peak-position analysis, the dark contrast was calculated as approximately 65.2%. On the contrary, the piezoresponse histogram of the PFM-image in Figure 3d shows the right-skewed distribution in the bright contrast, as shown in Figure 3f. The bright contrast was calculated as approximately 63.2%. These results and analyses provide clear evidence that the ZnO films on (+z) LiNbO_3_ formed the Zn-polar surface, while the O-polar surface of the ZnO films was produced on (-z) LiNbO_3_. Further, the PFM analysis supports the chemical-etching results.

**Figure 4:**
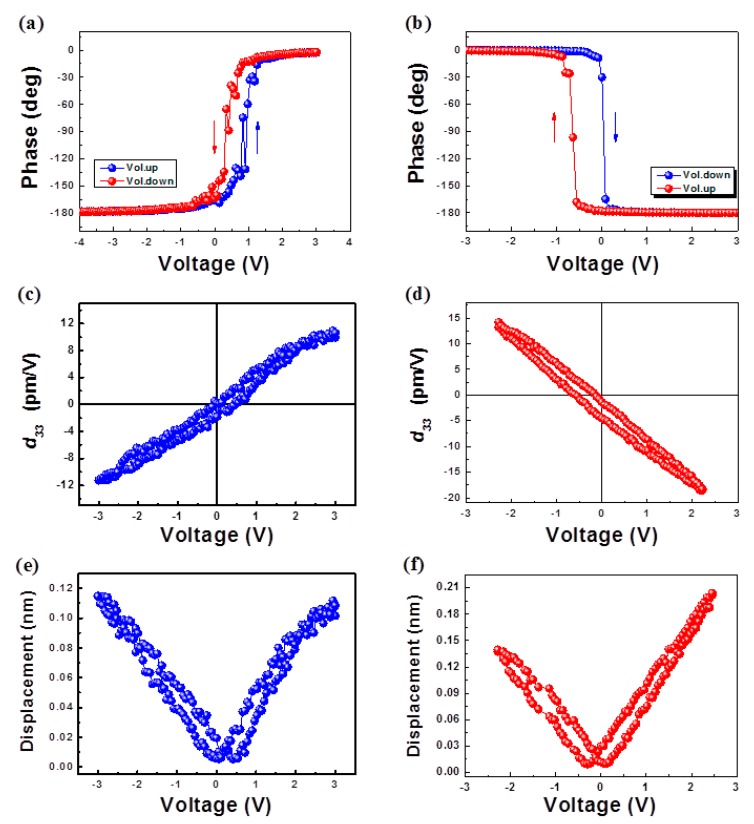
The hysteresis loop of the piezoelectric response for the ZnO films on (+z) LiNbO_3_ and (-z) LiNbO_3_, respectively. The phase-hysteresis loop of the ZnO films on (+z) LiNbO_3_ (**a**) and (-z) LiNbO_3_ (**b**), respectively. The effective piezoelectric-coefficient (*d_33_*) loop of the ZnO films on (+z) LiNbO_3_ (**c**) and (-z) LiNbO_3_ (**d**), respectively. The D-V butterfly loop of the ZnO films on (+z) LiNbO_3_ (**e**) and (-z) LiNbO_3_ (**f**), respectively.

Figure 4 displays the piezoelectric-response dependencies of the applied voltage for the ZnO films on (+z) LiNbO_3_ and (-z) LiNbO_3_ substrates. Figure 4a,b shows the piezoresponse histogram of the PFM-image recorded while the bias for the ZnO films was applied on (+z) LiNbO_3_ and (-z) LiNbO_3_, respectively. The 180° inversion at the coercive voltages with the hysteresis was observed in both of the ZnO films on (+z) LiNbO_3_ and (-z) LiNbO_3_. The phase change from −180° to 0° (Figure 4a) indicates a downward polarization (Zn-polar) for the ZnO film on (+z) LiNbO_3_, while the phase change from 0° to −180° (Figure 4b) revealed an upward polarization (O-polar) for the ZnO films on (-z) LiNbO_3_. A shift of the hysteresis loop was observed in both of the ZnO films on (+z) LiNbO_3_ and (-z) LiNbO_3_ substrates. These results can be explained by the imprint effect that is attributed to a built-in potential in the ZnO thin film [23]. Figure 4c,d exhibits well-shaped, effective *d_33_* loops for the ZnO films on (+z) LiNbO_3_ and (-z) LiNbO_3_, respectively. The effective *d_33_* values were 11.25 pm/V for the ZnO films on (+z) LiNbO_3_ and 15.94 pm/V for the ZnO films on (-z) LiNbO_3_, respectively. These values coincide with the previous reports about the piezoresponse of undoped ZnO films [24,25,26]. The typical D-V butterfly loop was obtained with displacement-maximum values of ~0.11 nm at ~3 V for the ZnO films on (+z) LiNbO_3_ and ~0.2 nm at 2.45 V for the ZnO films on (-z) LiNbO_3_, as shown in Figure 4e,f, respectively. The clear-opposite hysteresis characteristics and the nearly-symmetric loop shape of the piezoresponse indicate a polarity inversion [26] and high crystallinity of the ZnO films grown on the polarity-controlled LiNbO_3_. In addition, these results are in good agreement with the results of the HR-TEM, XRD, and chemical-etching analyses.

### 3.3. The Possible Mechanism

**Figure 5:**
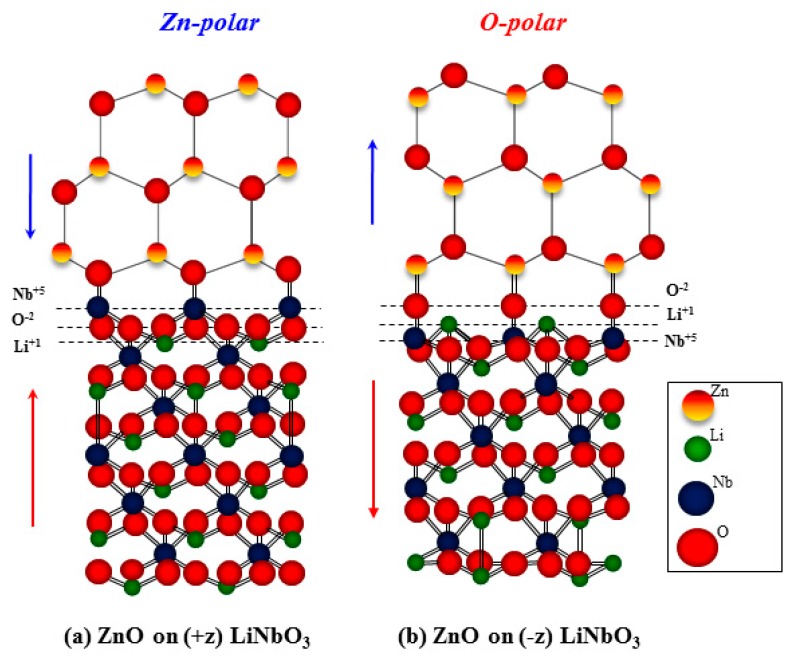
Schematics of the atomic arrangements of the ZnO films grown on (+z) LiNbO_3_ (**a**) and (-z) LiNbO_3_ (**b**), resulting in Zn-polar ZnO and O-polar ZnO, respectively.

LiNbO_3_ can produce a pyroelectric effect, for which the spontaneous polarization changes as a function of the temperature [27]. Given the previous results, the LiNbO_3_ pyroelectric effect was employed to demonstrate the growth mechanism of the ZnO films grown using the pulsed-laser deposition technique. To simplify the model, it was assumed that the screening from the external molecules and ions was completely removed by the cleaning process, or by the heating at the PLD-growth temperature of 600 °C. When the LiNbO_3_ crystal heated up from room temperature to 600 °C, the *P_s_* decrease resulted in the occurrence of further uncompensated screening charges on the surface. On the positive domain, the unscreened positive-polarization charge will generate a strong surface electric field. For the negative domain, the unscreened polarization charge is negative, causing the generation of an electric field directly toward the surface [28,29,30]. Figure 5a,b illustrates the schematics of the atomic arrangements of the ZnO films grown on (+z) LiNbO_3_ and (-z) LiNbO_3_, respectively. During the ZnO-film growth, the material plasma expanded away from the target surface as a plume that supplied the compensated charges to the surface of the LiNbO_3_ substrate, instantaneously. Consequently, the surface electric field formed by the unscreened polarization charges led to the absorption of the ionized atoms in ZnO with opposite polarity to surface polarization.

Once the neutralization was accomplished, the substrate no longer attracted the ionized atoms (Zn, O). Therefore, oxygen (O) atoms of Zn-polar ZnO formed the first layer on the (+z) LiNbO_3_ surface, and zinc (Zn) atoms of O-polar ZnO form the first layer on the (-z) LiNbO_3_ surface, and these did not change during the subsequent ZnO-epitaxial growth. Moreover, Saito annealed LiNbO_3_ at temperatures in the region from 500 to 1100 °C. In that case, the author proved the presence of a niobium (Nb) atomic layer on the positive surface by using coaxial impact–collision ion scattering spectroscopy (CICISS) to terminate the atomically smoothed surface [31]. 

Therefore, it is possible to conclude that the –O-Nb-O_3_ bridge is formed at the interface between the ZnO film and the (+z) LiNbO_3_ substrates, leading to the formation of the Zn-polar ZnO film, while the –Zn-O-Nb chemical bonding is formed at the interface between the ZnO film and the (-z) LiNbO3 substrate, leading to the formation of the O-polar ZnO film.

## 4. Conclusions

In summary, the polarity-controlled ZnO films were successfully grown on (0001) LiNbO_3_ using the pulsed-laser deposition technique. The cross-sectional image of the HR-TEM revealed that a clear interfacial layer with a well-ordered structure was obtained for the ZnO film that was grown on (+z) LiNbO_3_, while an unclear interface with a thin transition layer that was approximately 3 nm thick was formed for the ZnO film grown on the (-z) LiNbO_3_ substrate. According to PFM and chemical-etching analyses, the ZnO film grown on (+z) LiNbO_3_ was verified as Zn-polar ZnO, while the O-polar ZnO occurred on (-z) LiNbO_3_. Further, a possible mechanism of the interfacial atomic configuration between the ZnO films on (+z) LiNbO_3_ and (-z) LiNbO_3_ was suggested in the present paper. The two advantages of this new approach are as follows: 1) ZnO films with high crystalline quality can be grown without buffer layers on LiNbO_3_ due to a relatively low lattice mismatch. 2) The polarity of ZnO is directly and effectively controlled through the exploitation of the spontaneous polarity of the LiNbO_3_. Therefore, the results of the present study are valid for the polarity control of polar materials, thereby giving rise to a new approach for the modification of the material polarities in future optoelectronic and micromechanical devices.

## Supplementary Materials

The following are available online at https://www.mdpi.com/2079-4991/10/2/380/s1. Figure S1: (a) The 2θ-ω scan of the XRD spectra of the ZnO/(+z) LiNbO3 and ZnO/(-z) LiNbO3 thin films. (b) The ω-rocking curves of the ZnO/(+z) LiNbO3 and ZnO/(-z) LiNbO3 thin films. Figure S2 AFM images of the Zn-polar and the O-polar in the bulk-ZnO samples. (a) Zn-polar of the ZnO bulk before the etching. (b) O-polar of the ZnO bulk before the etching. (c) Zn-polar of the ZnO bulk after the etching. (d) O-polar of the ZnO bulk after the etching.
