# Supplementary material for: Polarity Control of ZnO Films Grown on Ferroelectric (0001) LiNbO3 Substrates without Buffer Layers by Pulsed-Laser Deposition
Source: Nanomaterials (Basel). 2020 Feb 22;10(2):380. doi: 10.3390/nano10020380 (PMC7075319; doi:10.3390/nano10020380)
Supplement: Supplementary file 1 [file nanomaterials-10-00380-s001.pdf]

Supplementary Information

Polarity control of ZnO films grown on ferroelectric (0001) $LiNbO_3$ substrates without buffer layers by the pulsed-laser deposition

Im Taek Yoon[1], Juwon Lee[1,*], Ngoc Cuong Tran[2] and Woochul Yang[2,*]

[1] Quantum Functional Semiconductor Research Center (QSRC), Dongguk University, 26 Phildong 3ga, Chung gu, Seoul, 100-715, Republic of Korea

[2] Department of Physics, Dongguk University, 26 Phildong 3ga, Chung gu, Seoul, 100-715, Republic of Korea

*Corresponding author: E-mail: wyang@dongguk.edu, juji99@dongguk.edu

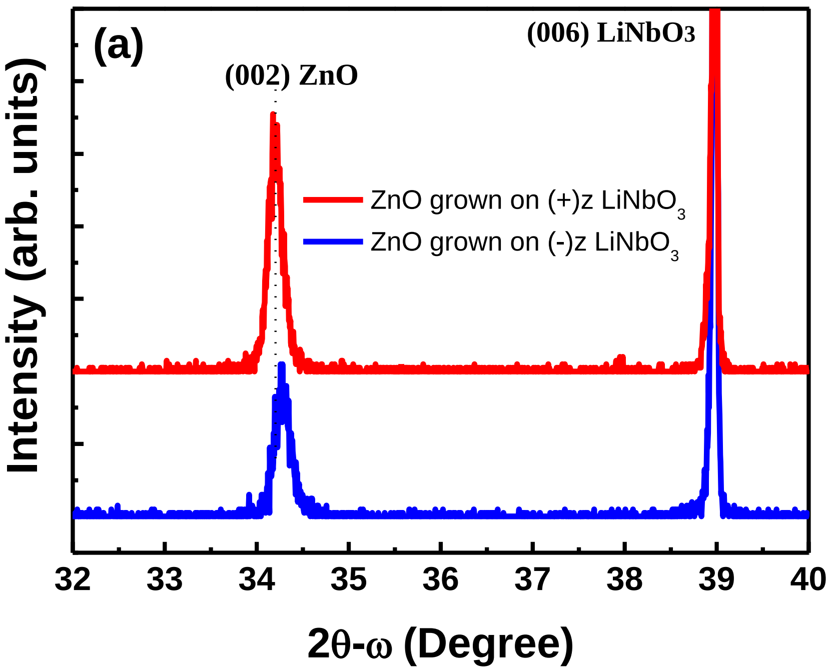

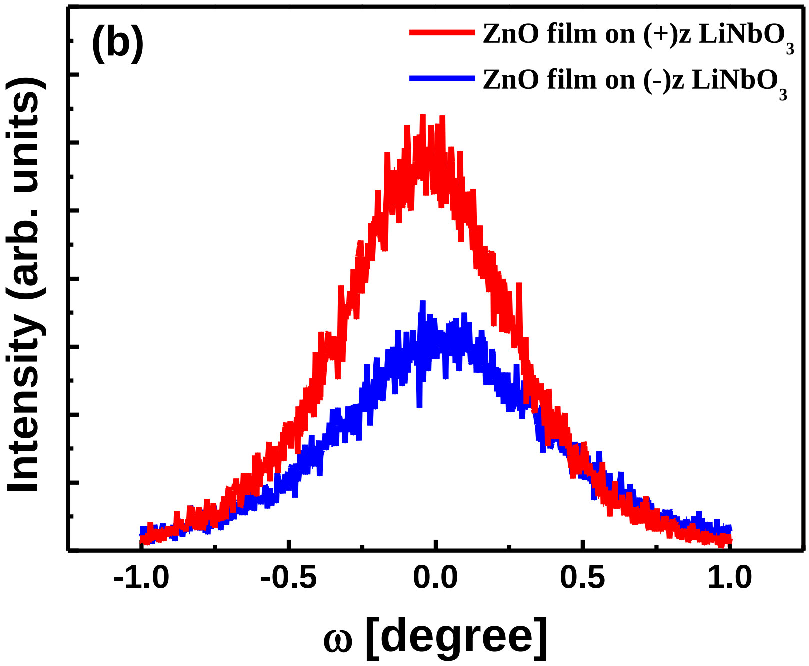

Figure S1 (a) The 2θ-ω scan of the XRD spectra of the ZnO/(+z) $LiNbO_3$ and ZnO/(-z) $LiNbO_3$ thin films. (b) The ω-rocking curves of the ZnO/(+z) $LiNbO_3$ and ZnO/(-z) $LiNbO_3$ thin films.

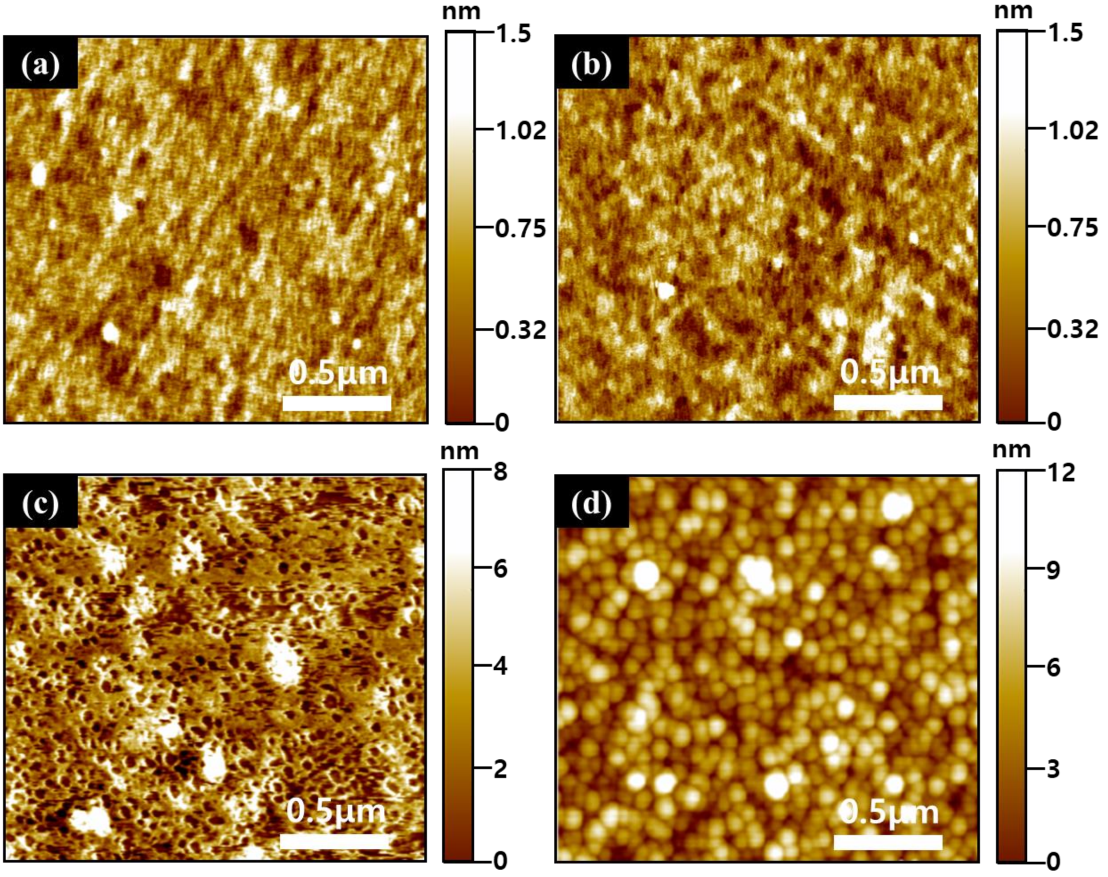

Figure S2 AFM images of the Zn-polar and the O-polar in the bulk-ZnO samples. (a) Zn-polar of the ZnO bulk before the etching. (b) O-polar of the ZnO bulk before the etching. (c) Zn-polar of the ZnO bulk after the etching. (d) O-polar of the ZnO bulk after the etching.
